# Effectiveness of Dry Needling and Ischaemic Trigger Point Compression of the Levator Scapulae in Patients with Chronic Neck Pain: A Short-Term Randomized Clinical Trial

**DOI:** 10.3390/jcm12196136

**Published:** 2023-09-22

**Authors:** Jorge Velázquez Saornil, Zacarías Sánchez Milá, Angélica Campón Chekroun, José Manuel Barragán Casas, Raúl Frutos Llanes, David Rodríguez Sanz

**Affiliations:** 1 NEUMUSK Group Research, Department of Physiotherapy, Facultad de Ciencias de la Salud, Universidad Católica de Ávila, 05005 Ávila, Spain; zacarias.sanchez@ucavila.es (Z.S.M.); jmanuel.barragan@ucavila.es (J.M.B.C.); raul.frutos@ucavila.es (R.F.L.); 2 Campus San Jerónimo Guadalupe, Universidad Católica de Murcia, 30830 Murcia, Spain; angelicacampon@gmail.com; 3 Facultad de Enfermería, Fisioterapia y Podología, Universidad Complutense de Madrid, 28005 Madrid, Spain; davidrodriguezsanz@ucm.es

**Keywords:** chronic neck pain, myofascial pain syndrome, manual therapies, trigger points, ultrasound elastography, physiotherapy techniques

## Abstract

Background: Chronic neck pain (CNP) may be associated with latent myofascial trigger points (MTrPs) in the levator scapulae (LS), which can be treated with ischemic compression (IC) and dry needling (DN). Variables and elastography changes are evaluated to compare the short-term efficacy of two treatments with DN. Methods: A randomized clinical trial is conducted with 80 participants in two groups: the DN group (*n* = 40) and IC group (*n* = 40). The duration is 12 weeks, and mechanical heterogeneity index, pressure pain threshold (PPT), and pain intensity are measured at baseline, immediately after, 48 h after, and one week after treatment. Results: Statistically significant changes were immediately observed between the two groups: PPT decreased in the DN group (*p* = 0.05), while it increased in the IC group. At 48 h and one week after treatment, these values increased in the DN group and remained higher than in the IC group. The heterogeneity index improved in both groups but more significantly in the DN group than in the IC group. Conclusions: In subjects with CNP who had latent plus hyperalgesic MTrPs in the LS muscle, DN outperformed IC in PPT, pain intensity, and mechanical heterogeneity index at 48 h and one week after initiating therapy.

## 1. Introduction

Chronic neck pain (CNP) is defined as localized pain that runs from the base of the skull to the first dorsal vertebra and is absent from the upper extremities [1]. A total of 79% of individuals will develop CNP at some point in their lifetime according to recent studies [2,3,4,5], making it a potentially serious musculoskeletal condition worldwide. According to studies, the frequency of CNP is 83% worldwide [1] and rises between the ages of 60 and 65 [2]. Without taking into account the various reasons associated with this type of pain, there has been a recent increase in the disability brought on by CNP in more developed nations, making it one of the top causes of disability worldwide [2,3,4,5]. CNP is more common in women than in men, and patients usually have recurring episodes. With the maximum incidence occurring in the third decade of life, the annual incidence rate typically varies from 20% to 55% [2,3].

Typically, 90% of cases are nonspecific, and the cause of the pathology is unknown in most populations [2]. Therefore, nonspecific CNP is characterized by the fact that it has no identified medical origin [1].

Due to its anatomical arrangement and design, the LS muscle may be the cause of chronic pain, as it has been proved that this muscle is constantly activated in many of the most common actions of daily life and can generate discomfort locally and even at a distance. This vital muscle in cervical statics also has an active role in the active component, as it must perform constant contraction in many movements since it can take a fixed point in the origin and its insertion; hence, it is a muscle responsible for CNP [1].

### 1.1. Myofascial Trigger Points (MTrPs) and Myofascial Pain Syndrome (MPS)

MTrPs are the most sensitive points within a tight band of muscle fibers (“taut band”) that hurt when compressed and may also be accompanied by local and/or referred pain. MTrPs are not known as myofascial pain syndrome; instead, they are the cause of myofascial pain syndrome when active and are thought to cause a variety of sensory, motor, and autonomic symptoms [3,4,5]. The most delicate areas on a tight muscle band that may be palpated are MTrPs, which can be either active or latent. When compressed, a patient can feel the presence of active MTrPs, which cause referred pain, restricted mobility, and weakening when at rest [6]. A local spasm response (REL) of the muscle fibers occurs when this trigger site is activated, causing autonomic and referred motor consequences. Latent MTrPs are inactive, do not produce pain on their own, and only hurt when they are touched. The necessary diagnostic criteria for the existence of an MTrP include range of motion restriction, the presence of a palpable tight band, identification of pain, and local and pressure-referred pain at the tight band’s nodule [7,8,9,10].

According to recent studies, MTrPs are the initial indicator of muscular overload. However, in addition to affecting the musculoskeletal system, this disorder also interacts with the visceral somatic system, affects the central and peripheral neurological systems, overproduces inflammatory mediators, and alters microcirculation [11,12,13,14].

Currently, the efficacy of IC and DN as MTrP treatments have been established [15,16,17,18]. The goal of DN is to carry out a treatment that has short-term benefits, reduces pain, improves joint ROM, and reduces disability in musculoskeletal disorders [3]. DN is an invasive technique performed by qualified healthcare professionals. Various deep DN methods exist, including the following:

Gunn intramuscular stimulation technique [6].

The most commonly used technique is Hong’s quick input–output method. REL has been demonstrated to disrupt motor endplate noise, producing an analgesic effect [6] and a fast depolarization of the affected muscle fibers, resulting in pain alleviation and increased range of motion [6,7].

In contrast to invasive techniques, noninvasive IC is one of the most recently used manual therapy techniques and is considered one of the most effective treatments for MPS [7]. A therapist applies pressure for 90 s on the MTrPs and progresses according to the patient’s tolerance. This affects the stimulation of mechanoreceptors that decrease pain signals and, thus, cause the normalization of the biomechanical properties of the muscle fibers [18].

### 1.2. Ultrasound Elastography

The imaging technique known as ultrasound elastography was initially introduced in the 1990s [19]. It has been improved and enhanced further in recent years to enable quantitative measurements of tissue stiffness. Elastography techniques make use of modifications in soft tissue elasticity brought on by particular pathological or physiological processes [19]. As a result, in diagnostic applications, elastography techniques can be utilized to distinguish between afflicted and normal tissue.

After DN, the mechanical heterogeneity index measures changes in the characteristics of muscle tissue that correspond to modifications in the state of MTrPs.

The aim was to measure several variables, assess changes at the elastography level, and compare the effectiveness of two potential short-term therapies for CNP.

## 2. Materials and Methods

In order to determine the short- and medium-term effectiveness of DN and IC therapy sessions for the treatment of LS muscle MTrPs in patients with CNP, a single-blind, randomized, two-group, assessor-blinded clinical trial was conducted. The four variables under investigation were: range of motion (ROM), pressure pain threshold (PPT), quality of life, and pain intensity. Measurements were taken before, immediately after, 48 h, and one week after the therapy session. Only the quality-of-life survey was administered before and after treatment. Each participant read, comprehended, and signed an informed consent form before taking part in the study. The results’ publication was also approved by the authors.

To achieve proper blinding, all study participants were randomly assigned before the session. Using the Epidat 3.1 program (www.sergas.es) (accessed on 1 September 2023), each subject was randomized to either DN or CI. Because he or she was not the one giving the intervention to the patients, the researcher collecting the study data was likewise blinded.

Prior to participating in the study, each participant signed informed permission forms, and the researchers agreed to uphold the Declaration of Helsinki, the relevant Personal Data Protection, and the Guarantee of Digital Rights regulations [20]. The study was approved by the Complejo Asistencial de Ávila research committee and registered on clinicaltrials.gov with the number NCT05776199. Patients were selected for this study from the FisioSalud Ávila Physiotherapy Clinic between October 2022 and March 2023. Previously, a traumatologist was required to diagnose the patient, and CNP clinical judgment was required. The lead investigator then noted the selection criteria for the people who were chosen and determined whether MMP was present in the LS muscle.

-Inclusion criteria were as follows: (I) the presence of a hypersensitive or hyperirritable point in the tension band; (II) patient reporting local or referred pain in the area of the latent MTrPs after mechanical stimulation; (III) the presence of a palpable tight band nodule in the LS muscle; (IV) patient reporting CNP for more than six weeks; and (V) signed informed consent forms.

-Exclusion criteria were as follows: (I) cervical surgery; (II) patient with neurological disorder; (III) age under 18 years; (IV) systemic or local infection in the cervical herniated disc’s cervical region; (VI) cervical herniated discs; (VI) intake or injection of anticoagulant or antiplatelet medication; (VII) the presence of needle phobia (belonephobia); or (VIII) pregnancy.

A two-group, randomized, single-blind clinical trial was conducted. This study examined the effectiveness of IC and DN therapies for latent MTrPs in the LS muscle in patients with CNP in conjunction with a sonoelastography examination. Additionally, the effectiveness of both treatments was hidden by the assessor.

**Figure 1:**
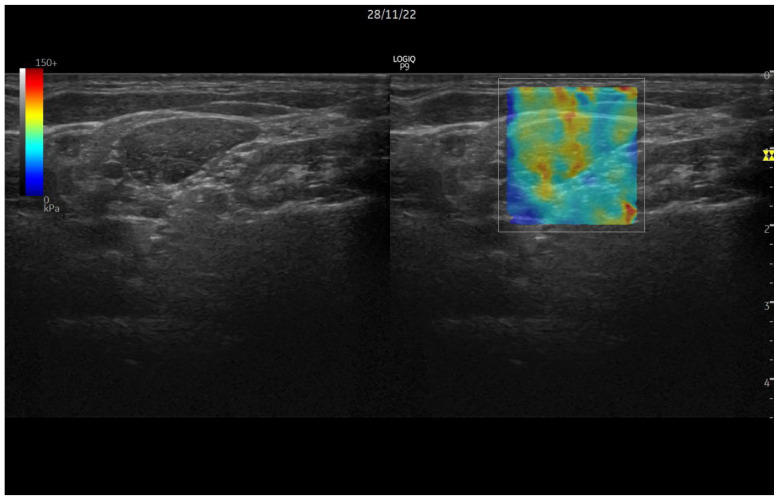
Sonoelastography ultrasound.

Figure 1 shows the sonoelastographic evaluation of the LE muscle.

### 2.1. Common Treatment Parts

First, latent MTrPs were found in the LS muscle in both groups. The four assessments performed on the participants during the intervention were before, after, 48 h after the intervention, and one week later. Each group received four measurements for each intervention: PPT, ROM, quality of life, and pain intensity.

### 2.2. Identification of Latent MTrPs

Without being aware of the treatment assignment, the same physiotherapist found latent MTrPs in the LS muscle when measurements were taken before and after the intervention. The researcher who executed both interventions attested to the presence of latent MTrPs. Latent MTrPs from the LS that were the most hyperalgesic were chosen and permanently marked with a cross [14,18]. A hyperirritable nodule of a taut band that was activated or caused pain upon palpation by digital compression and resulted in a limitation of joint range upon stretching was referred to as a latent MTrP [14,21].

### 2.3. Invasive Technique: DN Group (n = 40)

**Figure 2:**
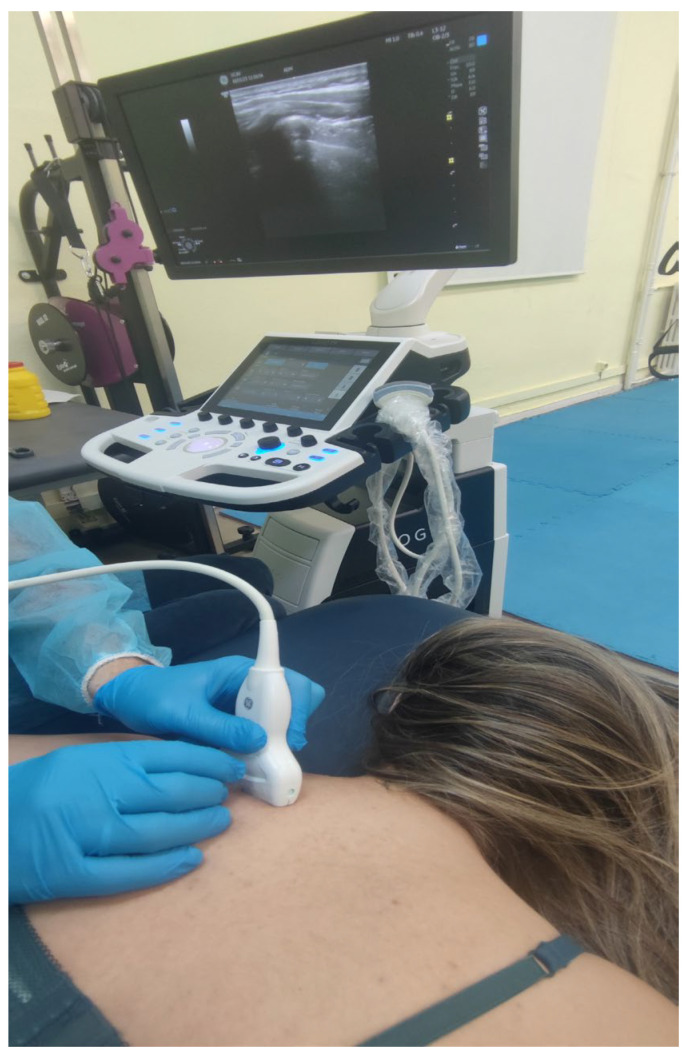
Dry-needling intervention.

In order to introduce a 0.25 × 60 mm stainless-steel needle, the patient was first positioned in a prone position by a physiotherapist in a sterile environment (Agupunt, Madrid, Spain). In order to evaluate and treat the most hyperalgesic latent MTrPs of the LS, which were defined as hyperirritable nodules of a taut band that were activated or produced pain upon palpation by digital compression and resulted in limitation of joint range upon stretching [22,23,24], they were chosen and marked with a cross with a permanent marker. The next step was to execute Hong’s input–output method over the MTrPs of the LS using an invasive ultrasound-guided method (Figure 2).

### 2.4. IC Group with Conservative Technique (n = 40)

A physiotherapist located the MTrPs of the LS muscle that were the most hyperalgesic before putting the patients in the prone position. His dominant hand’s thumb was then used to apply pressure until the patient’s pain threshold shifted from pressure feeling to pain for 90 s, and then he repeated the process three times [21].

### 2.5. Outcome Measures

Primary measures: The Visual Analogue Scale (VAS) was created to measure the severity of pain. It is made up of a 10 cm long horizontal line with a number 10 at the right end (signifying the worst pain) and a number 0 at the left [25]. Each person underwent this measurement before the therapy, just after it, 48 h later, and one week later.

Secondary measures:

Before and after each intervention, a CNP administered the Neck Disability Index (NDI) to each individual in order to gauge their level of disability [26]. This scale has been categorized as having the highest methodological quality with suggestion A (high level of development) [26]. Each of the 10 questions ranges in difficulty from 0 to 5, from least to most limited, with 6 understandable alternative responses. The result of each question added together and divided by the highest possible score multiplied by 100 yields the overall score, which is expressed as a percentage with a maximum of 100% [26].

Minimal limitation or impairment ranges from 0 to 20%;

Moderate limitation or disability is 21–40%;

Severe limitation or disability is 41–60%;

61–80% indicates disability.

More than 81% of the patients had the maximum functional limitations.

According to studies, DN and IC are both effective strategies for decreasing acute pain and improving ROM in MPS patients [22]. A cervical spine accelerometry instrument displays great reliability and modest variability in CNP patients during flexion and extension movements [27]. Patients were requested to bend their necks as far as they could while seated before being instructed to extend their cervical spine as far as they could. The device saved both measurements. Measurements from before and after the intervention were taken. The cervical range of motion was evaluated using a CROM goniometer [27]. The experiments were carried out in a spine kinesiology lab. Range of motion was measured using a CROM goniometer. A magnetic collar was fastened to the subject’s shoulders, and a goniometer was placed on their head; it was always positioned in the same orientation as the magnetic pole. During all measurements, the patients were sitting with their feet flat on the ground and their backs straight.

PPT stands for the lowest pressure or stimulation level at which a patient feels pain [28]. This measure was tested on previously selected latent MTrPs using a Wagner FORCE DIAL FDK 60 analogue algometer, which has great reliability for evaluating therapy efficacy in patients with MPS [29,30]. Each subject received it perpendicular to their latent MTrPs, and it built up pressure at a rate of 1 kg per second. Measurement was stopped when the subject indicated that he/she was in discomfort. Four measurements were made for every intervention: one before, one right after, one after 48 h, and one after a week. Once the recording process began, the subjects stayed in the same place. Measurements were made from the “neutral” position to the end range of motion in a particular plane, or during the “half-cycle” of a movement. Instructions were given before each measurement of the subject’s active range of motion, during which the examiner demonstrated the movement [31].

### 2.6. Data Analysis

A statistical application called IBM SPSS (version 27.0, IBM, NY, EE.UU) was used to conduct the analysis. To determine if a normal distribution existed, the Shapiro–Wilk test was initially employed to perform descriptive statistics on the control or independent variables. A descriptive statistical analysis was also used to test for the existence of a normal distribution. The homogeneity of the samples was evaluated. The parametric Student’s *t*-test for independent samples was used for quantitative variables having a normal distribution (*p* > 0.05). The nominal variable sample’s homogeneity prior to the intervention was examined using the chi-squared test. The nonparametric Mann–Whitney U test was applied for quantitative variables without a normal distribution (*p* = 0.05).

The numerous measurements taken before, during, after, and 48 h and one week after treatment in each group were examined using repeated measures analysis. Data collection pre-intervention, postintervention, 48 h after, and one week after made up the four stages of the within-subject component. The group variable, which had two levels (DN Group or IC Group), made up the second intrasubject component.

The Bonferroni method was used in a post hoc analysis to examine the variations in the groups’ measurements at different points in time. It was assessed whether the variables satisfied the sphericity requirement using Mauchly’s test (*p* > 0.05). The Greenhouse–Geisser correction was applied when a variable violated the Mauchly assumption of sphericity (*p* > 0.05).

## 3. Results

**Figure 3:**
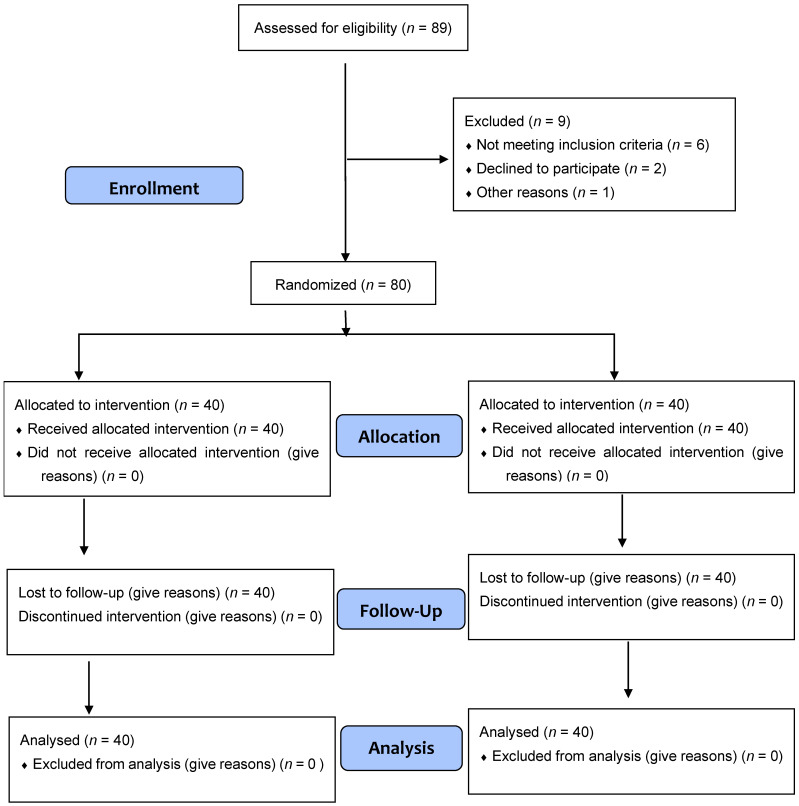
Flow diagram.

Out of the 89 people that were recruited, 9 were dropped from the study: one for pregnancy, two for not showing up for screening, and six for not having MTrPs at screening (Figure 3). Both intervention arms of the study experienced no side effects, and neither intervention arm experienced any losses because of withdrawal from the study or absence from screening.

Table 1 displays the descriptive statistics for the independent and control variables. Participants in the DN group and the IC group did not differ statistically significantly from each other on these control factors.

Table 2 contains the Student’s *t*-test for independent samples on the control variables.

Table 3 displays the descriptive statistics for the studied dependent variables.

When the dependent variables of VAS, algometry, and NDI were calculated using the linear technique of repeated measurements and the post hoc analysis was performed with the Bonferroni method, there were significant differences between the groups and the sample periods. There were no statistically significant changes between the groups and the time of consumption, as indicated by the ROM, one week after the intervention, with a result of *p* = 0.37 for both groups.

Table 4 presents the descriptive statistics for assessing pain intensity. Nonparametric tests were employed to compare results because it was found that this variable’s distribution was not normally distributed. According to the results of the Mann–Whitney U test, the IC group experienced more pain than the DN group did just prior to the intervention. A post hoc analysis after the treatment showed statistically significant differences (*p* 0.05) between the groups.

Table 5 details the LS muscle’s pain threshold in reaction to pressure. The linear repeated measures analysis revealed no statistically significant differences (*p* > 0.05) between the two groups at the pre-intervention time.

The flexion–extension range-of-motion test descriptive statistics are shown in Table 6. There were no statistically significant differences (*p* > 0.05) between the two groups when the measurements between the two groups were compared using the chi-squared test during the pre-intervention period. Furthermore, at one week, there were no statistically significant differences in ROM between the two groups (*p* > 0.05).

The descriptive statistics from the Oswestry quality-of-life survey are shown in Table 7. There were no statistically significant changes between the groups at the pre-intervention time in the exploratory analysis of this variable (*p* > 0.05). At one week after therapy, the quality-of-life measure showed improvements in both groups, while the DN group’s mean score was lower.

**Figure 4:**
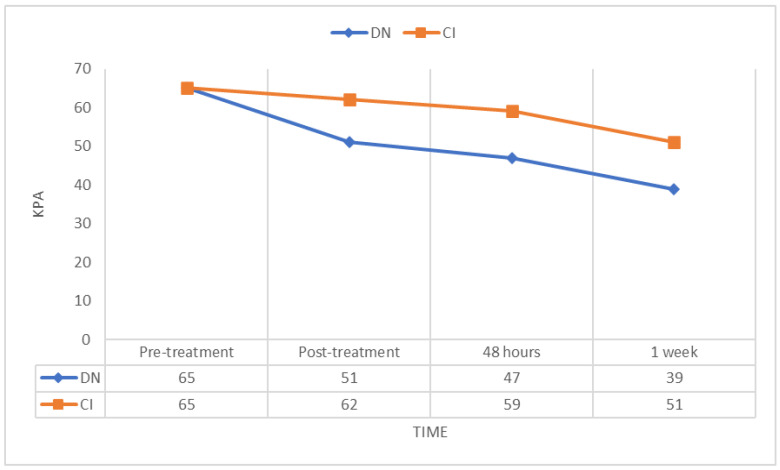
Mechanical heterogeneity index measure changes.

In addition, after 1 week, LE muscle stiffness at rest was lower in individuals who received DN than in those who received IC, as measured by the mechanical heterogeneity index (Figure 4). All other group differences in muscle stiffness were similar but not significant.

## 4. Discussion

In terms of pain intensity, ROM, PPT, and quality of life in the short term and one week after treatment in each intervention, this study is the first clinical trial to examine the efficacy of DN and IC on latent trigger points of the LS in patients with CNP. Because they instantly lessen pain and increase the range of motion, recent investigations have shown that IC and DN are successful therapies for patients with upper trapezius trigger points and CNP pain [32,33,34]. Additionally, both techniques have been shown to be effective adjuncts for treating chronic pain in CNP [34], lowering pain faster than sham or placebo techniques [35,36]. Other research found that neither approach has any positive benefits on pain intensity or ROM [37]. Our study’s findings demonstrate notable variations between the two approaches’ pressure pain thresholds, pain intensities, and quality of life, with DN outperforming IC both right away after the intervention and throughout the 1-week followup.

DN has been demonstrated to relieve pain right away after treatment in patients with musculoskeletal issues and is beneficial in treating CNP patients’ pain [32,33,34,35,36]. The needle causes neuromuscular damage, an inflammatory reaction, and hemorrhaging, and its effect contributes to reducing pain in patients by activating descending inhibitory pain mechanisms [38], reducing segmental nociceptive afferents from the MTrP, and acting on central sensitization [39], which is an amplification of the neural signal within the central nervous system that causes allodynia and hyperalgesia associated with the development and prevalence of chronic pain.

Dry needling involves some degree of discomfort for patients. This could be especially problematic when trigger points are treated in patients with comorbid conditions, such as fibromyalgia, characterized by a generalized increased sensitivity to pain. This may be caused by anomalous depolarization of the postsynaptic membrane of the motor plate, resulting in a localized hypoxic energetic crisis that is associated with sensory and autonomic reflex arcs maintained by complex sensitization mechanisms. For this reason, it is also possible to explain how certain disorders with hyperalgesia (such as fibromyalgia) may benefit in the long term even though they generate high pain after the intervention. Thanks to the theory of McPartland et al., these complex sensitization mechanisms can be interpreted [40].

These findings validate and complement research that demonstrated that all patients experience pain and hyperalgesia following the DN of a latent MTrP, which typically lasts less than 72 h [41]. It was demonstrated that IC is a highly effective method for treating MTrPs, resulting in instant pain alleviation [42]. Other research demonstrated that MTrP compression can regulate prefrontal cortex activity and potentially reduce pain in individuals with chronic CNP [43]. These studies concur with our findings that people receiving IC experienced instant pain relief compared to those in the DN group, indicating a short-term effect. However, the improvement did not improve with time.

Studies revealed that two sessions of DN and IC had equivalent effects on pain, ROM, and disability at the cervical level, with the DN group displaying better outcomes [44]. In a different trial when DN was applied to people with MTrPs in the upper trapezius muscle, the DASH disability scale improved [45].

The measures most frequently endorsed and used to gauge the severity of CNP-related disability worldwide are the Roland–Morris scale and the Oswestry CNP disability scale [46]. The latter was utilized in our study because it involved CNP. Our study’s findings revealed significant differences in the quality of life of both groups between pre- and postintervention, with the DN group experiencing a bigger improvement than the IC group.

Even though certain studies [47,48] have failed to find a statistically significant difference in pain intensity, other studies [48] showed that DN is one of the most effective ways to directly inactivate MTrPs, improve symptomatology, and relieve pain. According to studies, the local spasm response (REL) could lessen the loudness of the motor end plate and had a faster and long-lasting analgesic impact than if RELs were not produced [21]. This was brought on by the injured muscle fibers’ quick depolarization, which reduces discomfort and broadens the range of motion [42]. In order to obtain these results, our study, like those described before, used the Hong fast-in and fast-out technique up to the subject’s tolerance limit, which was 8–10 insertions.

According to research, MTrPs in the thoracic, lumbar, and trapezius muscles immediately see a reduction in PPT following a single DN treatment session [48,49,50,51,52]. Our results are consistent with past investigations, which discovered that, in the postintervention test, the DN group had a lower pressure pain threshold than the IC group. The PPT increased in these scores 48 h and one week after treatment, in accordance with a previous study that demonstrated that PPT levels rose when DN was conducted and may even be higher two days after the intervention [53,54]. Even after receiving two DN treatments, a study found that the elevation in the pain threshold under pressure rose and remained stable for two weeks.

The PPT rose after 48 h and remained the same after one week in our experiment with a single DN session. According to the studies cited above, the more local spasm responses that are obtained while taking the patient’s tolerance into account, the more potent and long-lasting the consequence. Llamas et al.’s research [51] on participants with cervical illness demonstrated that the PPT rose immediately following treatment and remained elevated 48 h later. However, in our study, the PPT was higher in the IC group compared to the DN group immediately following the intervention, but this improvement was not maintained 48 h or one week later.

According to our IC approach, which was used in earlier experiments [53], we administered the medication three additional times after the patient’s PPT changed from a pressure feeling to pain. A different study discovered that, in order to raise the PPT and exert force on the latent trigger points of the LS in the short term, applications should be performed for 60 s below the PPT without exceeding the patient’s discomfort threshold and for 90 s with elevated pressure, reaching the patient’s pain or discomfort threshold [54]. There are various restrictions on this study. First, the results’ applicability to other populations may be constrained by the fact that both groups received treatment from the same physiotherapist. Second, we chose a convenience sample that, while maybe modest, had a stronger effect and external validity based on prior studies with similar features. Thirdly, we cannot be positive that the results will hold up over time because they were only assessed after the treatment, at 48 h, and at one week. The investigation should, therefore, be conducted again in the medium term (from one to three months of development). These limitations should be considered in future studies.

## 5. Conclusions

In contrast to IC, DN successfully raised the quality of life, PPT, and pain intensity in patients with CNP on the most hyperalgesic latent MTrP locations of the LS muscle.

Immediately after the treatment, IC raised PPT, pain level, and quality of life. No statistically significant differences existed in ROM between the DN and IC groups.

## Figures and Tables

**Table 1:** Mean (M) and standard deviation (SD) of the control variables by group.

	Control Variables			
	DN Group *n* = 40		IC Group *n* = 40	
	M	SD	M	SD
Age	52.03	16.92	55.76	13.84
Weight (kg)	76.27	12.78	81.22	9.09
Height (cm)	179.03	4.01	180.88	3.79
Body mass index	24.37	3.52	23.96	3.28

**Table 2:** Student’s *t*-test for independent samples on the control variables.

	Student’s t	*p* < 0.05
Age	0.412	0.355
Weight (kg)	−0.796	0.196
Height (cm)	−0.985	0.137
Body mass index	−0.529	0.335

**Table 3:** Mean (M) and standard deviation (SD) of the dependent variables by group and time of sampling.

	DN Group *n* = 40		IC Group *n* = 40	
	M	DT	M	SD
VAS				
Pre	6.96	0.91	7.72	0.87
Post	6.68	1.79	3.56	2.21
48 h	4.49	0.96	4.32	1.19
1 week	4.31	1.85	4.21	4.58
ALGOMETRY				
Pre	5.26	0.68	5.35	1.32
Post	4.66	0.60	5.88	1.02
48 h	5.58	0.48	5.40	1.17
1 week	5.56	0.68	5.34	1.30
ROM				
Pre	0.33	0.51	0.43	0.53
1 week	0.07	0.37	0.14	0.37
Neck Disability Index				
Pre	22.17	6.49	23.86	5.84
Post 1 week	15.50	6.09	20.86	4.33

**Table 4:** Mean (M), standard deviation (SD), and *p* value of VAS by group and time of acquisition.

	Group	Pre	Post	48 h	1 Week
M	DN	7.41	7.09	4.60	4.20
	IC	8.07	3.87	4.80	5.00
SD	DN	0.82	2.37	0.98	1.26
	IC	1.03	2.06	1.20	1.81
*p* value (*p* > 0.05)	DN	0.292	0.987	0.001	0.001
	IC	0.271	0.001	0.001	0.002

**Table 5:** Mean (M), standard deviation (SD), and *p* value of algometry by group and time of measurement.

	Group	Pre	Post	48 h	1 Week
M	DN	3.97	3.96	4.61	4.61
	IC	4.53	4.91	5.02	5.09
SD	DN	0.89	0.81	0.89	0.89
	IC	0.96	0.89	0.99	1.23
*p* value (*p* > 0.05)	DN	0.405	0.036	0.031	0.051
	IC	0.725	0.022	0.501	0.806

**Table 6:** Mean (M), standard deviation (SD), and *p* value for ROM test results by group and time of collection.

	Group	Pre	1 Week
M	DN	0.20	0.10
	IC	0.27	0.10
SD	DN	0.41	0.25
	IC	0.45	0.25
*p* value	DN	0.082	0.182
	IC	0.086	0.182

**Table 7:** Mean (M) and standard deviation (SD) of the Oswestry questionnaire by group and time of taking the questionnaire.

	Group	Pre	1 Week
M	DN	21.09	14.80
	IC	23.08	21.25
SD	DN	6.07	3.05
	IC	5.26	5.93

## Data Availability

Not applicable.
